# Molecular length distribution and the formation of smectic phases

**DOI:** 10.3762/bjoc.5.65

**Published:** 2009-11-13

**Authors:** Nadia Kapernaum, C Scott Hartley, Jeffrey C Roberts, Robert P Lemieux, Frank Giesselmann

**Affiliations:** 1 Institut für Physikalische Chemie, Universität Stuttgart, D-70569 Stuttgart, Germany; 2 Department of Chemistry, Queen’s University, Kingston, Ontario, Canada

**Keywords:** bidispersity, liquid crystals, phase diagrams, smectic phases, structure and dynamics

## Abstract

The phase diagrams of two mixtures of chemically similar smectogenic mesogens strongly differing in molecular length were investigated. In these mixtures the nematic phase present in the pure short mesogen disappeared rapidly on the addition of the longer mesogen, while the smectic state was preserved. In the smectic state the smectic A phase was the much more stable phase as the smectic C phase disappeared quite rapidly as well. In these compounds the loss of the smectic C phase is accompanied by a decrease in smectic translational order and very small tilt angles. This leads to a concentration induced smectic C to smectic A transition. Thus smectic A seems to be the most stable phase to accommodate mesogenic molecules of substantially different length. These surprising results are of general interest for the understanding of the structure and dynamics of smectic phases, as the structure of these bidisperse smectics is signified by extensive out-of-layer fluctuations.

## Introduction

The classical (and highly successful) approach to systematically tailor liquid crystal materials for specific applications is the formulation of optimised mixtures consisting of several mesogenic compounds and non-mesogenic additives such as chiral dopants or UV-stabilizers. While the design of nematic mixtures is highly developed and widely applied, far less is known about the mixing of smectics and the particular effects thereof. In principle the mixing of different kind of mesogens can lead to a phase behaviour that differs completely from that of the pure compounds. This effect is even amplified in mixtures of mesogens with strongly differing molecular structure. In this paper we report a systematic study with mesogens strongly differing in molecular length.

We recently discovered that the electroclinic effect of a chiral smectic A* (SmA*) material (consisting of a phenylpyrimidine host and 4 mol % of a chiral atropisomeric dopant) was amplified by a factor of three after adding only 5% of another homologous phenylpyrimidine, the molecular length of which was about twice the length of the host molecule [1]. This remarkable electroclinic effect amplification stimulated a more general investigation of how the mixing of smectogenic homologues differing only in molecular lengths (and thus making the distribution of molecular length extremely bimodal) changes the structure and properties of the nematic and the SmA phases and the (possible) tilting transition to smectic C (SmC).

**Figure 1:**
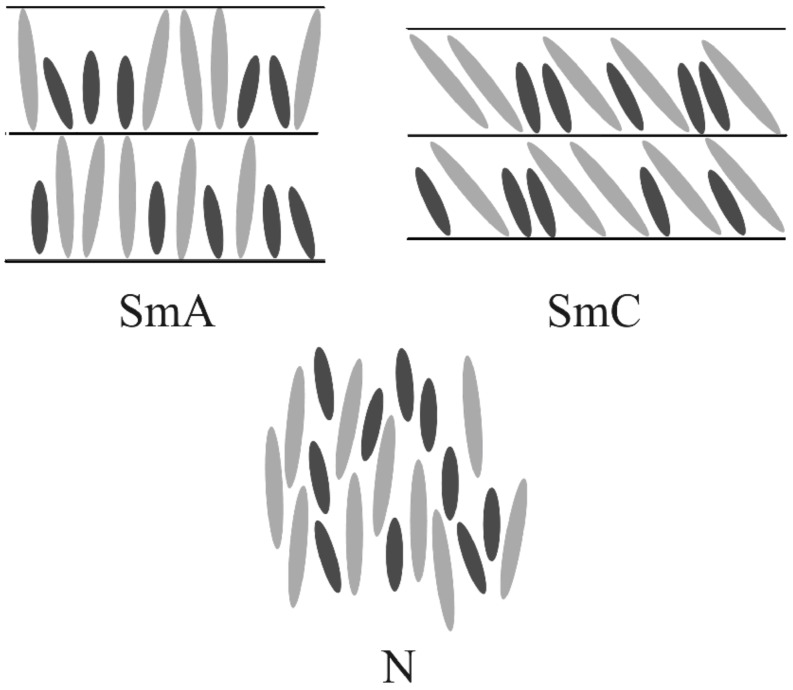
Structure of different liquid crystalline phases build with two kinds of hard spherocylinders with strongly differing lengths.

As a general first approximation calamitic mesogens are considered as rigid rods. This means they are treated as long and thin hard spherocylinders [2]. The justification for this rather crude approximation, which neglects the flexibility of the alkyl side chains, is the general observation that the thickness of e.g. a smectic A layer (as observed in X-ray diffraction) is only slightly smaller than the fully extended length of the constituting mesogenic molecules. In a naive model we now consider the liquid crystalline phase behaviour of mixtures of two types of these hard spherocylinders, which exhibit the same diameter but their lengths are differing by a factor of two. In this naive model, the nematic phase is expected – due to the absence of translational order – to be the ideal liquid crystalline (LC) phase for accommodating molecules of substantially different lengths. On the other hand the smectic phases – due to their layer structure – seem to be unsuited to accommodate molecules of different lengths. Comparing SmC and SmA, the SmC phase might be better since it allows different tilt angles to fit different molecular lengths into a smectic layer with fixed spacing (Figure 1).

The actual results received in this study are indeed completely reverse to the naive picture drawn above. The bimodal length distribution of smectogenic molecules favours the formation of non-tilted SmA phases at the expense of nematic or tilted SmC phases.

## Results and Discussion

**Scheme 1:**
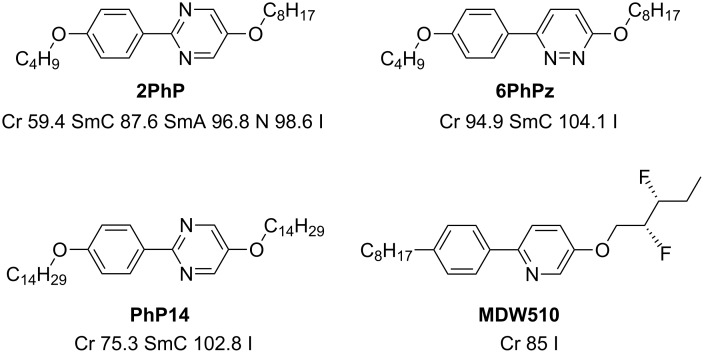
Chemical formulas and phase sequences of the mesogens **PhP14**, **6PhPz** and **2PhP** and the chiral dopant **MDW510**.

**Figure 2:**
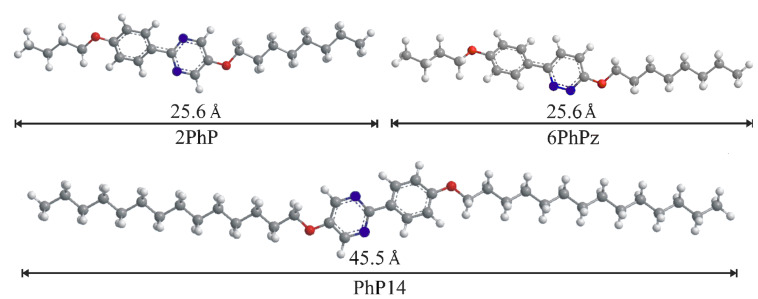
Molecular structure of the mesogens **PhP14**, **6PhPz** and **2PhP** differing in molecular lengths by a factor of approximately two. The molecular lengths were determined from the most extended conformers, after optimizing their energy using molecular modelling with MOPAC/AM1.

Scheme 1 and Figure 2 show the liquid crystalline materials we used in this experimental study. As the long component we chose the phenylpyrimidine **PhP14** (2-[4-(tetradecyloxy)phenyl]-5-(tetradecyloxy)pyrimidine) [1], where the aromatic core is substituted symmetrically with two alkoxy chains each with 14 methylene units. It exhibits a molecular length of 45.5 Å. For the short compound we used either the phenylpyrimidine **2PhP** (2-[4-(butyloxy)phenyl]-5-(octyloxy)pyrimidine) [1] or the phenylpyridazine **6PhPz** (6-[4-(butyloxy)phenyl]-3-(octyloxy)pyridazine) [3–4]. Both were asymmetrically substituted with two alkoxy chains with four and eight methylene units, respectively. Their molecular length is 25.6 Å. This leads to a difference in lengths of a factor of the order of two.

**Figure 3:**
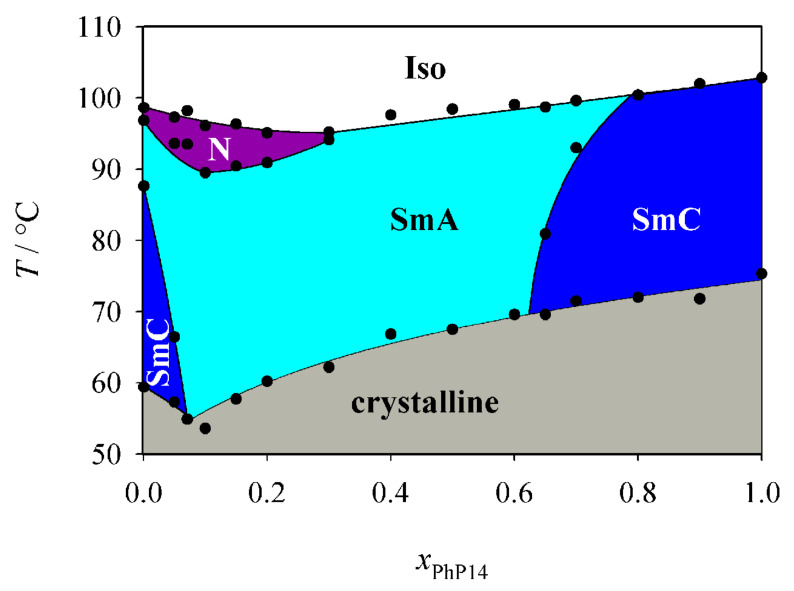
Phase diagram of the system **2PhP/PhP14**. Over a broad temperature and concentration range only the SmA phase is stable.

First we investigated the phase diagram of the system **2PhP**/**PhP14** which is shown in Figure 3. This phase diagram shows completely different behaviour from that expected from the naive model. In particular we observed no indication of destabilization of the smectic state. We could even observe a eutectic point at *x*_PhP14_ = 0.075, where the temperature range of the smectic state is broadened. The nematic state on the other hand disappears rapidly with increasing mole fraction *x*_PhP14_ and is already lost at a mole fraction *x*_PhP14_ of 0.3. In the smectic state SmA turns out to be the much more stable phase. SmC disappears quite rapidly and SmA is the dominating phase in the phase diagram even though SmC is the dominating LC phase of **2PhP** and the only LC phase of **PhP14**. Taking into account that the average alkyl chain length increases with increasing *x*_PhP14_, this behaviour is to some extent analogous to the well known mesomorphism in homologous series where nematic is the dominating mesophase for short-length homologues, SmA for medium-length homologues and SmC for longer homologues (see e.g. [1]).

**Figure 4:**
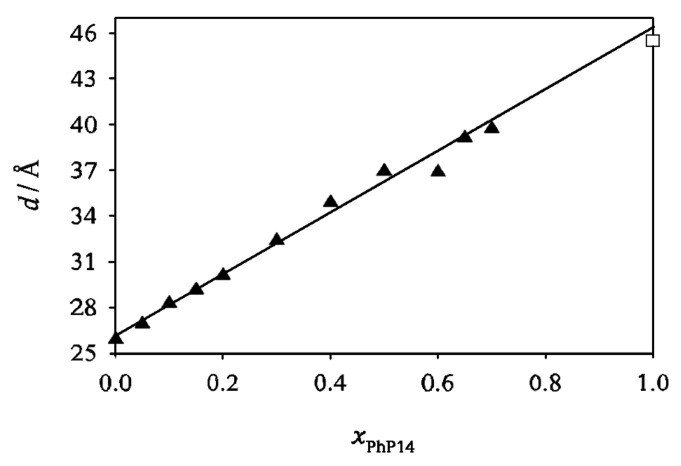
The layer spacing in the SmA phase at *T* = *T*_c_ vs mole fraction for the system **2PhP**/**PhP14** (filled triangles) and calculated value of the molecular length for pure **PhP14** from molecular modelling (open square).

For all mixtures small angle X-ray scattering (SAXS) measurements were performed. Figure 4 shows the layer spacing in the SmA phase at *T* = *T*_c_ in dependence on the mole fraction. A linear correlation between the layer spacing and the mole fraction is found. This linear dependence shows that the system **2PhP**/**PhP14** follows the Diele additivity rule [5]. It says that the layer spacing of a mixture can be calculated as: *d*_Mix_ = *d*_A_ *x*_A_ + *d*_B_ *x*_B_, where *d*_Mix_, *d*_A_ and *d*_B_ denote the layer spacings of the mixture, the pure compound A and the pure compound B, respectively and *x*_A_ and *x*_B_ the mole fractions of compound A and B, respectively. From this equation the layer spacing of a hypothetical SmA phase of pure **PhP14** can be estimated. The extrapolated value of 46.4 Å corresponds quite well to the value of 45.5 Å we obtained for the extended length of the **PhP14** molecule from molecular modelling studies (see Figure 2). For **2PhP** the experimental *d*-value from SAXS (25.9 Å) also agrees very well with the extended length of the molecule (25.6 Å). This agreement between the experimental *d*-values and the extended molecular lengths for both pure compounds justifies to a certain extent the application of the spherocylinder model in these cases.

**Figure 5:**
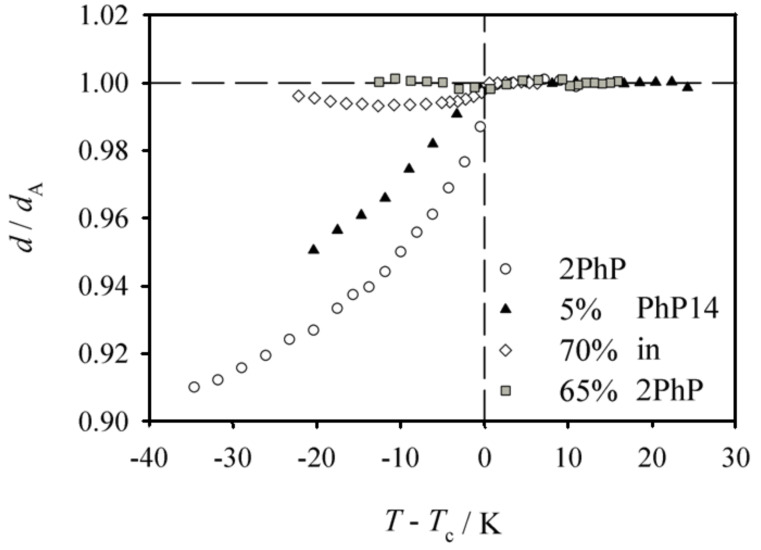
Reduced layer spacing for the mixtures which exhibit SmA and SmC phases in the system **2PhP**/**PhP14**. Pure **2PhP** shows quite normal layer shrinkage of 7% at *T*−*T*_c_ = −20 K (open circles). The addition of 5% of **PhP14** reduces the layer shrinkage to only 5% at *T*−*T*_c_ = −20 K (filled triangles). For the mixtures with excess of the long homologue **PhP14** the layer shrinkage is dramatically reduced. The 70%-mixture exhibits a layer shrinkage of only 1% at *T*−*T*_c_ = −20 K and for the mixture with 65% no layer shrinkage below the SmA to SmC phase transition could be observed at all.

**Figure 6:**
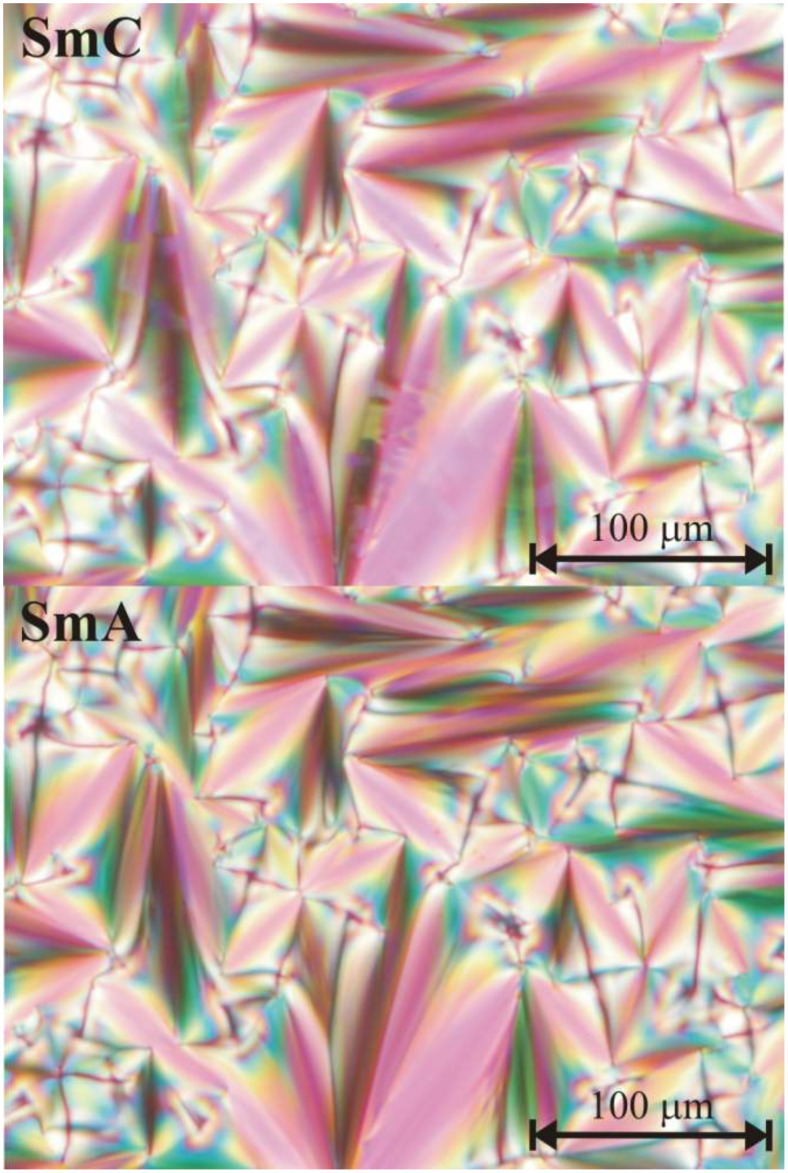
Textures of the mixture with 65% **PhP14** in **2PhP** as observed in the polarizing microscope. The upper part shows the broken fan-shaped texture of the SmC phase at *T* = 75 °C. In the lower part the fan-shaped texture of SmA is observed at *T* = 86 °C.

Figure 5 gives an overview of the layer spacings of pure **2PhP** and of the mixtures with 5%, 65% and 70% **PhP14** in **2PhP**, respectively. The reduced layer spacing (calculated from the measured layer spacing divided by the layer spacing of the SmA phase) is plotted vs the temperature difference *T*−*T*_c_ relative to the phase transition temperature from SmA to SmC. The pure compound **2PhP** shows a ‘common’ behaviour of the layer spacing with significant layer shrinkage due to the molecular tilt in SmC of about 7% at *T*−*T*_c_ = 20 K. In the mixture with 5% **PhP14** the layer shrinkage in the SmC phase is reduced to only 5%. Very small layer shrinkage of only 1% was found for the mixture with 70% **PhP14**. And for the mixture with 65% **PhP14** no layer shrinkage at all could be found, although by polarizing microscopy the broken fan-shaped texture of a SmC phase was clearly observed (see Figure 6).

**Figure 7:**
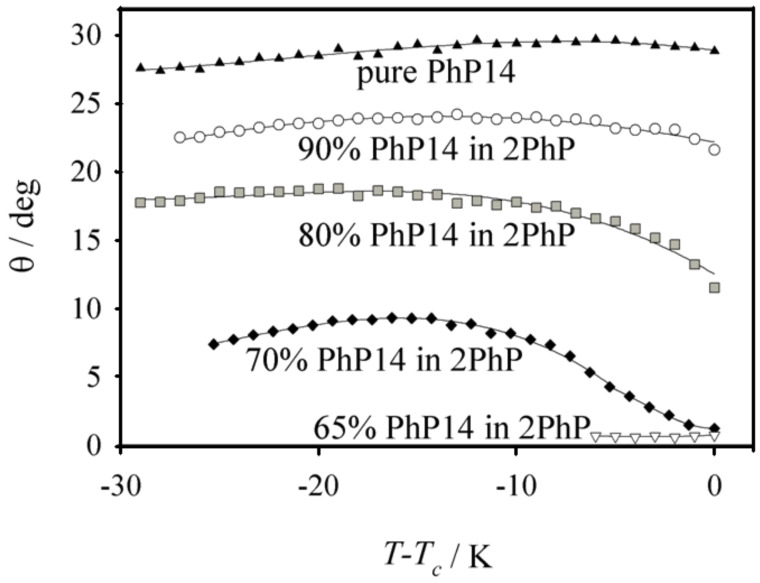
Tilt angle vs temperature difference to the phase transition temperature for pure **PhP14** (filled triangles), 90% (open circles), 80% (gray filled squares), 70% (filled diamonds) and 65% (open triangles) **PhP14** in **2PhP**. The tilt angle is reduced successively with increasing mole fraction of the short component **2PhP**. In this system a concentration-induced phase transition from SmC to SmA occurs.

To gain a deeper understanding of these smectic phases the optical tilt angle of the mixtures was measured in the corresponding ferroelectric SmC* state [6] (see Figure 7) after addition of 4 mol % of the chiral dopant **MDW510** ((*R*,*R*)-2-[4-(octyloxy)phenyl]-5-(2,3-difluorohexyloxy)pyridine) [7–8]. The pure compound **PhP14** exhibits the highest tilt angles, with a quite regular value of about 27°. The addition of more and more of the longer molecules reduces the SmC-tilt stepwise until only a SmA phase is left. This system thus shows a concentration induced SmC to SmA phase transition and therefore opens the possibility to design SmC phases with very small tilt angles [9]. These tilt angles are in good agreement with the tilt angles calculated from the X-ray layer shrinkage after: θ = cos^−1^(*d*_C_/*d*_A_). This correlation between the optical and X-ray tilt angles shows that, even if the layer shrinkage is very small, the mixtures do not necessarily exhibit the so-called ‘de Vries-type’ behaviour [10].

For a deeper insight into the quality of molecular ordering inside the smectic layer structure the translational order parameter Σ [11] of the SmA phases was determined by a method previously described in [12]. The translational order parameter Σ gives a measure for the quality of smectic layering. It is defined as the amplitude of the density wave arising from the 1D-periodic smectic layer structure.

**Figure 8:**
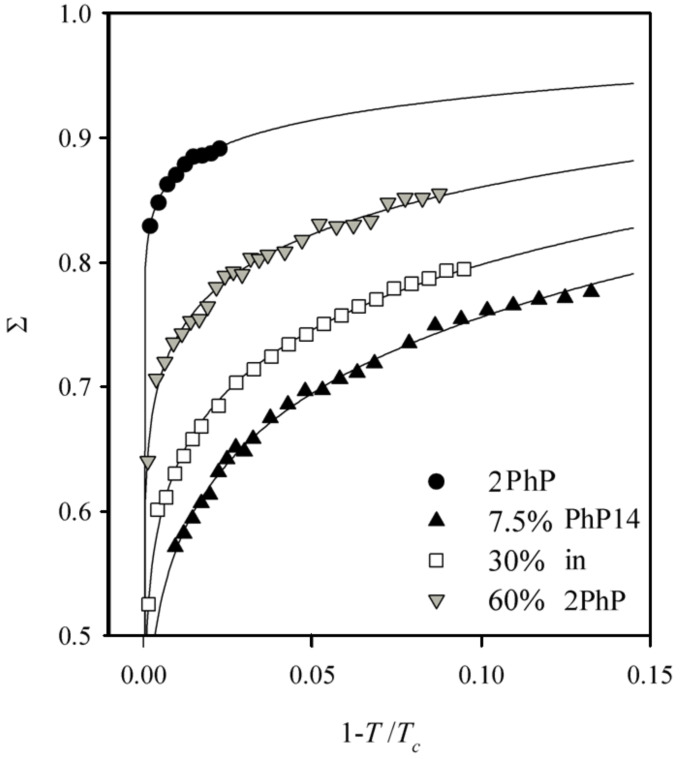
The translational order parameter Σ in the SmA phase is plotted vs the reduced temperature 1 – *T*/*T*_c_ for the pure component **2PhP** and the mixtures with 7.5%, 30% and 60% **PhP14** in **2PhP**, respectively. The pure **2PhP** shows the highest order parameters of about 0.9. The value of Σ is reduced after the addition of 7.5% of **PhP14** to about 0.7 and increases again slowly by the addition of more **PhP14** for the mixture with 30% **PhP14** until it reaches a value of 0.8 for the 60% mixture.

Figure 8 shows the smectic order parameters Σ of the SmA phase for the pure component **2PhP** and for the mixtures with 7.5%, 30% and 60% **PhP14** in **2PhP**, respectively. Pure **2PhP** forms a SmA phase with a high degree of smectic order. It exhibits a smectic order parameter of about 0.9. By adding a small amount of the other component the translational order is considerably reduced. For the mixture with 7.5% **PhP14** (the eutectic mixture, see Figure 3) Σ is in the range of about 0.7 and further increases on the addition of more of **PhP14**. It shows a value of about 0.75 for the 30% mixture and finally reaches a value of about 0.8 in the 60% mixture. The smallest value for Σ was observed at that point in the phase diagram where the SmC phase is lost. And the re-entering of the SmC phase into the phase diagram is preceded by a recovery of the smectic order.

To check whether these findings can be generalized, we investigated another phase diagram of two strict SmC mesogens with a length ratio of 1:2. The mixing of two strict SmC mesogens should lead to a phase diagram where only SmC phases emerge.

**Figure 9:**
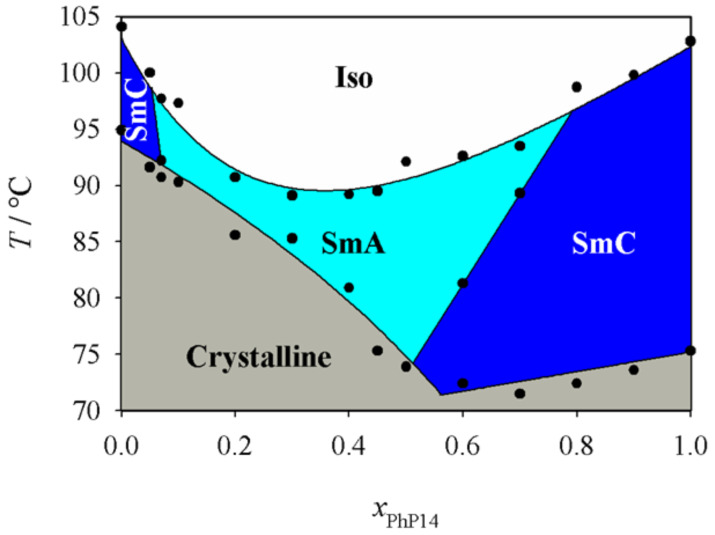
Phase diagram of the system **6PhPz**/**PhP14**. Over a broad temperature region only the SmA phase is stable, although the two pure compounds exhibit only SmC phases.

Figure 9 shows the phase diagram of the system **6PhPz**/**PhP14**. This phase diagram is very similar to the one of the system **2PhP**/**PhP14**. Although the two pure compounds show just SmC phases, the SmC phases disappear rapidly with increasing *x*_PhP14_ and SmA replaces SmC. However, the stability of the smectic state is preserved again.

**Figure 10:**
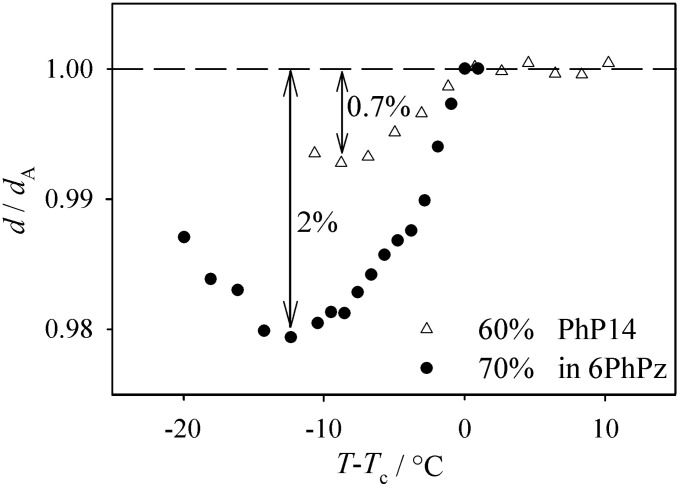
Reduced layer spacing vs temperature difference to the phase transition temperature for the mixtures with 60% and 70% **PhP14** in **6PhPz**. The layer shrinkage is only 2% for the 70% mixture and 0.7% for the 60% mixture, respectively.

The reduced layer spacing of the mixtures with 60% and 70% **PhP14** in **6PhPz** can be seen in Figure 10. Both mixtures show small layer shrinkage of only 2% for the 70% mixture and 0.7% for the 60% mixture, respectively. This small layer shrinkage might also be related to small tilt angles in the corresponding SmC phases. The smectic order parameters Σ of the mixtures could not be compared with those of the pure compounds, as the method described in [12] only applies to SmA phases whereas the pure components exhibit SmC phases only.

## Conclusions

Our investigations on two different phase diagrams of mesogenic molecules with chemically similar cores but with length ratios in the order of 2:1 led us to the following general results:

- When the short-length compound exhibits a nematic phase, the nematic phase disappears quickly with increasing mole fraction of the compound with greater molecular length.
- Surprisingly, the temperature range of the smectic states is preserved. It even becomes broader in some cases. Nevertheless the quality of smectic layering is lowered.
- We observed that over a broad temperature range the SmC phase is completely lost even though SmC is the dominating phase in the pure compounds. Instead of SmC, now the non-tilted SmA phase temperature range is broadened.
- In the regimes before SmC phases disappear, the mixtures show exceptionally small tilt angles (maximum tilt < 10° over about 20 K).

These results open pathways to a systematic design of interesting new low-tilt SmC materials.

To learn more about the ordering in smectic phases we compared our results with the work of Koden et al. [13]. They investigated several bidisperse mixtures of molecules with strongly differing molecular cores, but the same molecular length. In their mixtures they observed the same behaviour as in our mixtures. The nematic phases disappeared rapidly, as well as the smectic C phase, while the smectic A phase was the only stable phase over the whole phase diagram. They also found a dramatic decrease of the tilt angle in the remaining SmC phases, until the SmC phases disappeared at a concentration-induced phase transition to SmA.

These quite counterintuitive results are of general interest for the understanding of the structure and dynamics of smectic phases. Several theoretical approaches have been made to predict the behaviour of bidisperse mixtures [14–18]. They analyzed the stability of smectic A, nematic and isotropic phases by theoretical models of the Onsager-type in dependence on the composition of mesogens with different length and aspect ratios. They indeed predicted the occurrence of smectic phases in bidisperse mixtures for certain length and aspect ratios. In all these theoretical works however a stabilization of the nematic state at the expense of the smectic state was found. This is contradictory to the experimental findings, as our findings showed a stabilization of the smectic state while the nematic phase disappeared completely. The existing theories thus do not describe these results correctly. Furthermore there is no theoretical work on the influence of bidispersity on the balance between SmA and SmC.

**Figure 11:**
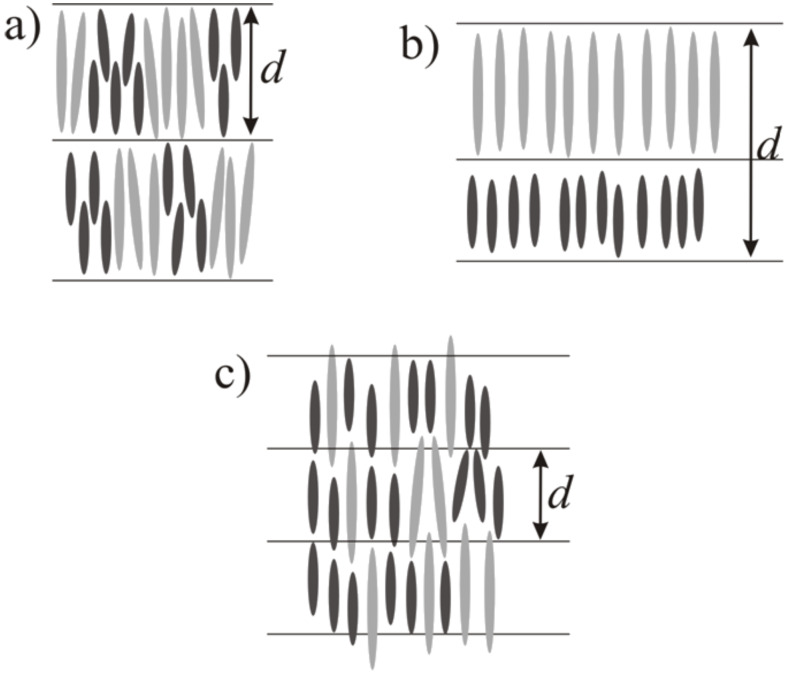
Schematic sketches of different models for smectic A ordering of bidisperse mesogens. Different models are: a) Intra-layer segregation, b) Inter-layer segregation or c) Out-of-layer fluctuations. After [5,18].

In the existing theoretical approaches different models for the ordering in smectic A phases of molecules of substantially differing lengths are presented (Figure 11).

One possibility is to fill the space by nanosegregation of long and short mesogens. This segregation can be of the intra-layer (Figure 11a) or inter-layer (Figure 11b) type. Both options however are entropically unfavourable. In the intra-layer type all molecules are organized within one layer. Demixing of the two kinds of mesogens occurs locally inside the layers. Furthermore the layer spacing of such a smectic phase would – for all mole fractions – correspond to the length of the longer molecule (Figure 11a). However, in our SAXS-measurements we observed a layer spacing which varied linearly with the mole fraction and it was always smaller than the length of the long molecule. In the second kind of nanosegregation – the inter-layer type – the two kinds of molecules demix and each of them forms their own layers. The ‘layer spacing’ observed by SAXS (cf. repeating unit) for this kind of smectic phase would correspond to the added lengths of the two molecules (Figure 11b). As the experimentally found layer spacing is always smaller than the long molecule, this also cannot be the correct explanation.

The last possibility is a more dynamic picture, where the space is filled by out-of-layer fluctuations (Figure 11c). The long molecules organize themselves in the layers formed by the short molecules by out-of-layer fluctuations. The layer spacing of this kind of smectic A phases would be in between the lengths of the two molecules, while the quality of smectic ordering, e.g. the smectic order parameter Σ, would be essentially lowered in comparison to the pure compounds. The experimental findings for both the layer spacing and the smectic ordering are in complete agreement with this model (see Figure 4 and Figure 8).

With all this results we thus believe that out-of-layer fluctuations are the most realistic model to describe the structure of bidisperse smectics. This also explains the strong influence of bidispersity on the balance between SmA and SmC. Since SmA phases might tolerate out-of-layer fluctuations much more easily, a stabilization of SmA at the expense of the SmC phase might occur. Therefore, the structure of bidisperse smectics is signified by extensive out-of-layer fluctuations.

## Experimental

Compounds 2-[4-(tetradecyloxy)phenyl]-5-(tetradecyloxy)pyrimidine (**PhP14**) [1], 6-[4-(butyloxy)phenyl]-3-(octyloxy)pyridazine (**6PhPz**) [3] and (*R*,*R*)-2-[4-(octyloxy)phenyl]-5-(2,3-difluorohexyloxy)pyridine (**MDW510**) [7] were synthesized according to published procedures and shown to have the expected physical and spectral properties. The liquid crystal 2-[4-(butoxy)phenyl]-5-(octyloxy)pyrimidine (**2PhP**) was obtained from a commercial source. X-Ray scattering experiments were performed with Ni-filtered CuK_α_ radiation (wavelength 1.5418 Å). Small angle scattering data from unaligned samples (filled into Mark capillary tubes of 0.7 mm diameter) were obtained using a Kratky compact camera (A. Paar) equipped with a temperature controller (A. Paar) and a one-dimensional electronic detector (M. Braun). For polarized optical microscopy a Leica DM-LP polarizing microscope with an Instec HS1-i hot stage was used. The optical tilt angles θ were determined by polarizing microscopy on samples aligned in rubbed nylon/ITO coated glass cells with a spacing of 1.6 μm (AWAT PPW, Poland). To enable ferroelectric switching of the tilt direction [19–20] in the achiral mixtures, 4 mol % of the chiral dopant **MDW510** was added to receive chiral SmC* phases. The measurements of θ were taken at a field strength *E* of 12.5 V μm^−1^ as half the rotation between the two optical extinction positions corresponding to opposite signs of *E*. A Netzsch DSC-204 Phoenix instrument was used for differential scanning calorimetry analyses at a scan rate of 5 K min^−1^.
